# Developing a Suitable Model for Water Uptake for Biodegradable Polymers Using Small Training Sets

**DOI:** 10.1155/2016/6273414

**Published:** 2016-04-21

**Authors:** Loreto M. Valenzuela, Doyle D. Knight, Joachim Kohn

**Affiliations:** ^1^Department of Chemical and Bioprocess Engineering, Research Center for Nanotechnology and Advanced Materials “CIEN-UC”, Pontificia Universidad Católica de Chile, Vicuña Mackenna 2860, Macul, 7820436 Santiago, Chile; ^2^Department of Mechanical and Aerospace Engineering, Rutgers, The State University of New Jersey, New Brunswick, NJ 08854-8087, USA; ^3^New Jersey Center for Biomaterials, Rutgers, The State University of New Jersey, 145 Bevier Road, Piscataway, NJ 08854, USA

## Abstract

Prediction of the dynamic properties of water uptake across polymer libraries can accelerate polymer selection for a specific application. We first built semiempirical models using Artificial Neural Networks and all water uptake data, as individual input. These models give very good correlations (*R* ^2^ > 0.78 for test set) but very low accuracy on cross-validation sets (less than 19% of experimental points within experimental error). Instead, using consolidated parameters like equilibrium water uptake a good model is obtained (*R* ^2^ = 0.78 for test set), with accurate predictions for 50% of tested polymers. The semiempirical model was applied to the 56-polymer library of L-tyrosine-derived polyarylates, identifying groups of polymers that are likely to satisfy design criteria for water uptake. This research demonstrates that a surrogate modeling effort can reduce the number of polymers that must be synthesized and characterized to identify an appropriate polymer that meets certain performance criteria.

## 1. Introduction

Degradable materials are very important in fabricating biomedical devices. After implantation, they do not need to be removed; rather, under ideal conditions, the implant site repairs itself while the device is resorbed [1]. In comparison, nondegradable materials often need to be surgically removed after their purpose has been achieved, thus subjecting the patient to a second surgery that potentially exposes them to more complications [2]. Degradable devices can be used in a broad range of applications such as vascular stents, vascular bypass grafts, bone fixation devices, and soft tissue replacement scaffolds [3].

Degradable biomaterials have a wide range of requirements depending on the particular clinical application. Parameters such as chemical structure, composition, porosity, and device geometry determine surface and bulk properties of an implant, and thus, they are critical to the selection of the material [4].

One important characteristic of degradable biomaterials is their water uptake versus time, as it is crucial for the determination of how long a polymeric device will reside in the body before erosion leads to the ultimate removal of the device from the implant site [5]. Water uptake affects degradation, swelling, mechanical [6], and adhesive properties [7]; also it determines drug stability [8], drug release profile [9], and biological response [10].

Current methods used to measure water uptake versus time are labor intensive and time consuming. Depending on the polymer, water uptake can take days to weeks to equilibrate [11]. There are potentially very large libraries of polymeric biomaterials, which make it impractical to measure these parameters experimentally for each polymer. For example, a virtual library of about 40,000 polymethacrylates has been described by Kholodovych et al. [12]. This library would clearly be too large for each polymer to be characterized individually by experimental methods.

Computational modeling is a useful tool to minimize the number of experiments needed to characterize a polymer library [13]. Costache et al. [14], Gubskaya [15], and Le et al. [16] published reviews that include the most relevant models currently available for important parameters in biomaterials such as glass transition temperature (*T*_*g*_), Young's modulus, air-water contact angle, water uptake, and degradation. Serna et al. (2008) built a model of equilibrium water uptake for 12 aromatic polyamides with very similar levels of water uptake (13.9%–19.1%). They found correlations between the amidic hydrogen charge and the water uptake [17].

Although empirical mathematical modeling has been successfully used to model water uptake for different polymers, all models require parameters that can only be obtained through experimentation. Fick's diffusion [18], anomalous Fickian diffusion [19], dual-stage Fick's diffusion [20], power law [18], Weibull equation [21], Langmuir theory [22], and concentration-dependent diffusion coefficient model [23] have been used. Modeling of hydration at the molecular level has been demonstrated using parameters such as free volume redistribution frequency [24], Radial Distribution Functions (RDFs) [25], 3D atomic density maps known as spatial distribution functions [26], and angular distribution functions [27]. Furthermore, from MD simulations, water absorption has been predicted for a single polymer system [28–30].

Prior works by Kholodovych et al. [12, 31], Smith et al. [32–35], Gubskaya et al. [36], and Ghosh et al. [37] showed that it is possible to build computational models of polymer properties for an entire library based upon experimental data for a small subset. In these studies, a polymer library is explored using a combined experimental and computational approach, looking for polymers that fulfill a series of design criteria to be suitable for specific applications. Smith et al. [33, 34] developed semiempirical models using molecular descriptors obtained from two-dimensional polymer structures (i.e., the descriptors were independent of the polymer conformation). These models were able to predict fibrinogen adsorption within experimental error in 38 out of the 45 polymers and rat lung fibroblast proliferation in 41 out of 48 polymers. Pearson correlation coefficient values for these predictions were 0.54 ± 0.12 and 0.54 ± 0.09, respectively. Gubskaya et al. [36] calculated descriptors from relaxed three-dimensional polymeric structures obtained from Molecular Dynamics (MD) simulations of tetramers in vacuum and implicit water. In this work, Decision Tree Analysis and ANNs were used to predict fibrinogen adsorption with a Pearson coefficient of 0.67 ± 0.13. The incorporation of three-dimensional descriptors led to important improvements in comparison with previous semiempirical models, increasing the average Pearson correlation coefficient from 0.54 ± 0.12 to 0.67 ± 0.13.

One of the challenges of biomaterials is the change of their interactions and properties over time [38]. However, all aforementioned models study and predict individual values for each polymer. They do not consider dynamic properties that may change over time. Even Le et al. (2013), who built predictions of phase behavior over time, developed the model using each experimental value as a single input, without considering how the phase behavior changes over time [39]. Previously, we built ANN models to accurately predict drug release over time on a family of terpolymers [40] using molecular descriptors. In this study, we develop and compare models for water uptake over time, first using all individual data separately and then using a global parameter for this property.

Our research has two objectives: (i) the development of computational models for water uptake versus time based upon experimental data from a small subset of polymers in a library and (ii) the application of these models to predict water uptake for an entire library of polymers. The main challenge of this research is to model and predict properties that change over time with particular kinetics using a small set of experimental data. As a model system, a library of L-tyrosine-derived polyarylates was used. Kohn and collaborators used this library to discover promising lead polymers for several medical applications [41], such as bone pins [42], hernia repair devices, and an antibacterial sleeve that protects recipients of implanted cardiac assist devices from potentially life-threatening infections [43].

This library, consisting of A-B-type copolymers having an alternating sequence of a diphenol and a diacid [41], was obtained by copolymerizing 14 tyrosine-derived diphenols with 8 aliphatic diacids in all possible combinations resulting in 112 distinct polymers. Changes in polymer backbone or pendent chain length affect polymer properties such as *T*_*g*_ and hydrophobicity. In this study we investigate the effect of polymer backbone and pendent chain on the water uptake profiles of polymer films.

## 2. Materials and Methods

### 2.1. Materials

A subset of the L-tyrosine-derived polyarylates was synthesized as described previously by carbodiimide-mediated solution polycondensation of a diphenol and a diacid at room temperature [44].

#### 2.1.1. Nomenclature

DTR = desaminotyrosyl-tyrosine alkyl ester: R = methyl (M), ethyl (E), iso-propyl (iP), butyl (B), iso-butyl (iB), sec-butyl (sB), hexyl (H), octyl (O), dodecyl (D), benzyl (Bn), 2-(2-ethoxyethoxy)ethyl (G).

HTR = hydroxyacetic acid-tyrosine alkyl ester: R = ethyl (E), hexyl (H), octyl (O).

### 2.2. Experimental Methods

#### 2.2.1. Film Processing

Polymer films were compression molded and annealed at 5–10°C above *T*_*g*_ for 20 h before incubation, as described previously [11].

#### 2.2.2. Water Uptake

Water uptake was obtained for the selected polymers from the L-tyrosine-derived polyarylates combinatorial library (Table 1) using ^3^H-labeled water, as described previously [45]. Briefly, films 1 cm in diameter were incubated in ^3^H-radiolabeled water (0.2 *μ*Ci/mL) at 37°C. After 6 h and 12 h and 1, 2, 3, 4, 7, 14, 21, 28, 35, and 42 days, samples were removed from the vial, rinsed with distilled water, blotted dry, and dissolved with 3 mL of tetrahydrofuran (THF) (VWR) and 12 mL of liquid scintillation cocktail (LSC) (Ecolite). Radioactive counts were measured using a scintillation counter (Beckmann 6500), and water content (*M*_3_H_2_O__) was calculated using a calibration curve. Water uptake (WU) was calculated as the water content relative to the original dry weight (*M*_sample_):

(1) $$\begin{array}{c} WU\left(\;\%\;\right)=100\cdot \frac{M_{3_{H_{2}O}}}{M_{sample}}. \end{array}$$

Table 2 lists the estimated values for equilibrium water uptake from the experimental measurements; both this parameter and individual water uptake experimental points were used to build surrogate models for water uptake.

### 2.3. Computational Methods

**Figure 1:**
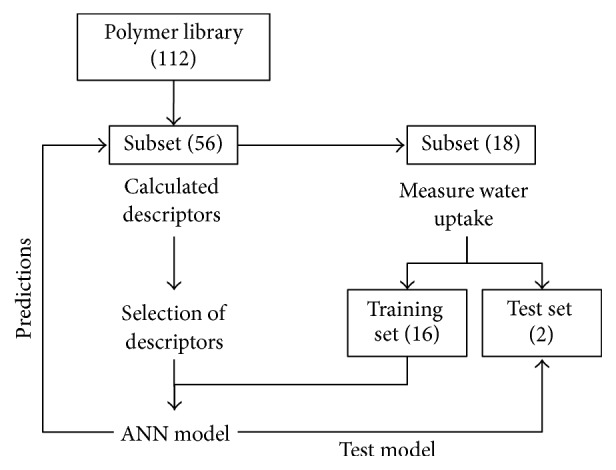
Scheme of experimental method for surrogate models of water uptake.

The data-mining package WEKA (Waikato Environment for Knowledge Analysis) [46] was used in this study. The methodology can be summarized in the following steps (Figure 1):

- Polymers were characterized using two-dimensional (2D) descriptors [32] and three-dimensional (3D) descriptors [36].
- Descriptors to build the model were selected using correlation based feature selection (CFS), expectation-maximization (EM) cluster analysis, Decision Tree Analysis, and linear regression.
- Either all water uptake experimental data points over time or equilibrium water uptake was used to build the model using ANNs, using 10% for testing and the rest for training.

#### 2.3.1. Descriptors

The descriptors in this study include “2D” descriptors based on the chemical structure of the polymers [32] and “3D” descriptors based on the chemical structure of the polymers in implicit water or vacuum incorporating polymer conformation [36]. Two-dimensional descriptors for the entire library of 112 polymers were obtained by Smith et al. [34], using the basic molecular structure derived from the chemical formulae and both the Molecular Operating Environment (MOE, Chemical Computing Group Inc.) [47] and the Dragon (Milano Chemometrics and QSAR Research Group) [48] commercial software packages. Three-dimensional descriptors were obtained by Gubskaya et al. [36] for 56 polymers from the polyarylate library. Descriptors were obtained by the Dragon commercial software package using the 3D structures of the tetramers after structure minimization and 1 ns of MD simulations using MacroModel v.8.5 (Schrödinger) [49] commercial package with the generalized Born/surface area implicit solvent model [50] and the OPLS-all atom force field [51]. Although 3D descriptors obtained from tetramers do not capture the realistic structure of large *M*_*w*_ polymers, they include very important information about their structure, which allows building more accurate models, as shown previously by Gubskaya et al. [36]. Similarly, other authors had previously used monomers [52] or less than 5 repeating monomeric units [53] to obtain molecular descriptors.

#### 2.3.2. Descriptor Selection

Starting with 2,272 descriptors taken from Gubskaya et al. [36] and Smith et al. [32], a correlation based feature selection (CFS) was used to reduce the dimensionality of the descriptors for each parameter in study. CFS is a function available in WEKA that evaluates the worth of a subset of attributes (descriptors) by considering the individual predictive ability of each feature along with the degree of redundancy between them. As a result, it selects a subset of attributes that are highly correlated with the parameter while removing irrelevant, redundant, and noisy attributes [54]. A genetic search algorithm was used in conjunction with the CFS, allowing a parallel search of the attribute space and avoiding local optima.

For each model, expectation-maximization (EM) [46] cluster analysis was employed to categorize the polymer property of study (i.e., water uptake and equilibrium water uptake) into three classes (i.e., low, medium, and high). When analyzing all data points for water uptake, both time and water uptake values were included in the cluster analysis.

The most significant descriptors were selected using a J48 Decision Tree [55], selecting descriptors that correctly partition the water uptake values and equilibrium water uptake according to the EM cluster analysis. Because Decision Tree Analysis cannot represent relationships between continuous variables, an additional descriptor was selected by linear regression, that is, the highest weight on the linear regression, for the full training set and the experimental values of water uptake. Time was also included as a descriptor for water uptake with all data points.

#### 2.3.3. Artificial Neural Networks

Linear models are insufficient to capture the complexity of the structure-property-relationships between polymer structure and water uptake profiles. Specifically, we observed that water uptake does not yield a simple correlation with the hydrophilic factor, as defined by Todeschini et al. [56] and calculated by Smith et al. [32].

Several authors have shown that an ANN model provides more accurate predictions than a linear model [57–62]. A multilayer perceptron (MLP) was used to build ANN models for each parameter with the three descriptors selected as explained in Section 2.3.2. Two hidden layers (nodes) were used. Output nodes were unthresholded linear units [46]. Backpropagation by gradient descent was used as MLP learning method. All input variables were scaled to the unit interval while the learning rate and the momentum applied for updating the weights were 0.3 and 0.2, respectively. Training time was set on 1,000 epochs, which showed to be enough for model convergence. To perform cross-validation, 10% of data was separated as test set in each model, in all possible combinations. Randomization of the initial weights and shuffling of the training data were performed by varying the seed for the random number generator. The model obtained with each seed represents a local optimum, based on the initial weights. Thus, running enough seeds and selecting the best model among them would allow finding the global optimum. For the present models, a hundred ANN models were obtained with different seeds, from which the best model in terms of root mean squared error for the training set was selected.

## 3. Results and Discussion

### 3.1. Descriptors Selection

Table 3 summarizes the descriptors selected for both models. One 3D descriptor and five 2D descriptors were selected for the model for all time points; two 3D descriptors and one 2D were selected for the model of WU_eq_. 2D descriptors include nCt, hydrophilic factor, SMR_VSA6, GGI3, MATS3m, and C-003. nCt is the number of tertiary carbon atoms (sp3). The hydrophilic factor is calculated from the number of hydrophilic groups (-OH, -SH, and -NH) of the molecule [63] and it was previously used to predict biological response on this polymer library [34]. SMR_VSA6 is a descriptor of subdivided surface area, based on accessible van der Waals surface area of each atom [64], and type of descriptor used before to predict fibrinogen adsorption of this polymer library [35]. GGI3 is a topological charge descriptor; similar topological descriptors have been used to predict biological response on polymethacrylate surfaces [37]. MATS3m is a Moran autocorrelation descriptor, which describes the level of correlation between molecules, and it has been used to study protein interactions [65]. C-003 is the number of CHR3 molecular subfragments, an atom center fragment; it gives information about structural motifs important for the molecular shape and it was used before to predict fibrinogen adsorption on polymethacrylate surfaces [37].

3D descriptors include G2m and R8p+ in vacuum and Mor25m in water. G2m is a WHIM descriptor, which captures relevant 3D information about molecular size, shape, symmetry, and atom distribution with respect to invariant reference frames [66]. WHIM descriptors were used to predict fibrinogen adsorption on polymethacrylate surfaces [37]. R8p+ is R-GETAWAY descriptor, which accounts for the local aspects of the molecule such as branching, cyclicity, and conformational changes [67].

Mor25m is a 3D-MoRSE descriptor, which provides structural information of the molecules in the space [68], and it has been suggested that this information is related to the free volume of molecules [69, 70] and, thus, responsible for the ability of the polymer to uptake water. 3D-MoRSE and GETAWAY descriptors have been also correlated with the tendency of a molecule to be solvated by water, measured by the hydrophilic index Hy [71], as defined by Todeschini and Consonni [63]. These types of descriptors encode relevant information of this polymer library that gives information of several physical and chemical processes such as water uptake and even in fibrinogen adsorption as discussed by Gubskaya et al. [36].

### 3.2. Model for Water Uptake

Results in Table 4 show that correlation coefficient is not the best indicator of model accuracy. Both models present high *R* ^2^ of training set (>0.92). However, the model using all data presents only 17% or less of predictions within experimental variability, for training and test sets, while the model for WU_eq_ is able to predict 67% for training and 50% for test, within experimental variability.

Results of cross-validation have to be analyzed very carefully when using all data points, because they are not independent of each other. In that case, it is likely to select for cross-validation data that belong to polymers for which there is a large data set in the training set. Thus, depending on how the cross-validation set is selected, different results will be obtained.

**Figure 2:**
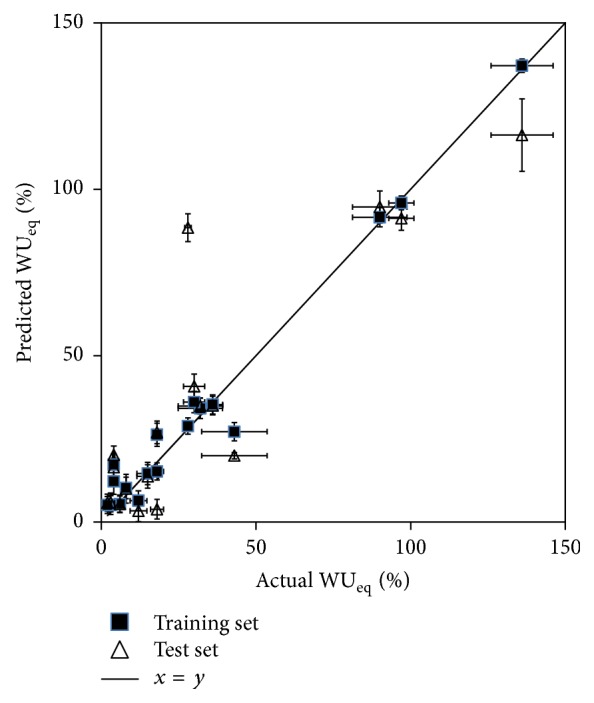
Prediction versus experimental values for WU_eq_ for polymers as part of training (■) and test (△) sets. Black line represents *x* = *y*. Values are presented as mean value ± SD of predictions (*y*-error) ± SD of experimental values (*x*-error).

On the other hand, the model for WU_eq_ obtains its values from several experimental measurements of each polymer after its water content is equilibrated. This gives more representative and reliable experimental data, and it captures more information than single points at the same time of incubation. With this, cross-validation that in this case includes only independent values, considering all possible combinations of leave-two-out (10%) of experimental values, gives accurate predictions in 50% of the cases from test sets, and WU_eq_ was correctly classified as high, medium, or low according to the EM cluster analysis previously done, in 83% of the cases. With this, predictions accurately represent the relative order in water uptake of the polymers studied (Figure 2 and Table 4).

This result is less accurate than predictions of simple physical behaviors such as *T*_*g*_ [14], but it is much more accurate than predictions of more complex processes such as fibrinogen adsorption [34, 36], cell growth [34], and gene delivery efficiency [72], where the Pearson coefficient for these models was below 0.77.

### 3.3. Predictions of Water Uptake over Rest of the Library

**Figure 3:**
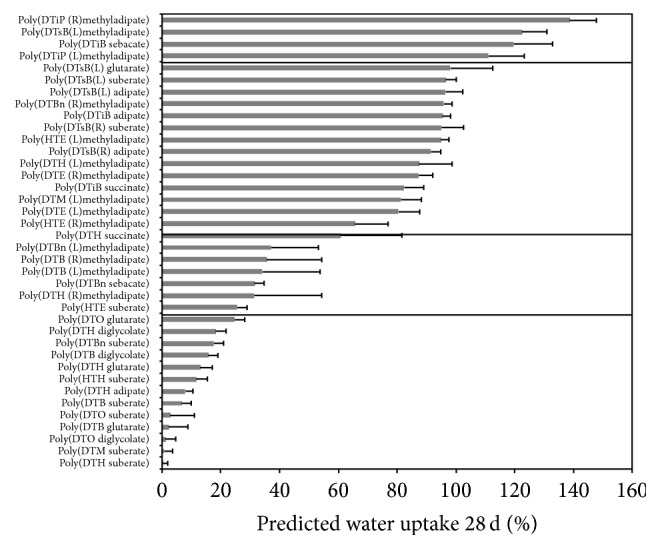
Predictions of equilibrium water uptake over the remaining 38 polymers of the polymer library. Values are presented as mean value ± SD of the predicted value for each training/test set combination. Polymers are ordered from highest to the lowest water uptake predicted values. Solid lines separate areas of very high, high, medium, and low water uptake polymers.

For each training and test set selection, predictions of equilibrium water uptake were made for the rest of the 56-polymer library. As Figure 3 shows, the model predicts low levels of water uptake for polymers containing DTM (0%–14%), DTO (0%–25%), and HTH (5%–14%) (with the exception of methyladipates); low to intermediate levels of water uptake for polymers containing DTBn (18%–34%) and DTE (35%–37%) (with the exception of methyladipates), glutarate (0%–37%), suberate (0%–26%) (with the exception of DTsB), and sebacate (5%–32%) (with the exception of DTiB); intermediate levels of water uptake for succinate-containing polymers (13%–61%); medium to high levels of water uptake for DTiB-containing polymers (82%–120%); high levels of water uptake for DTiP methyladipates (111%–139%); and widely ranging levels of water uptake for DTH (0%–87%), adipate (5%–96%), and methyladipate (31%–139%) polymers. It also predicts that all DTB polymers have low values of water uptake (less than 36%) and only high values of water uptake for DTsB polymers (92%–135%); it predicts low values of water uptake (10%–26%) for HTE polymers (with the exception of methyladipate) and predicts low levels of water uptake for diglycolate polymers (0%–18%).

Some of these predictions would be expected directly from the chemical structure of the polymers, but others would not be easily expected. For example, all DTO polymers would have low water uptake, which is expected from the long pendant chain (8 carbons), while the DTH polymers, with only one carbon less than the DTO, would have water uptake levels from low to medium.

### 3.4. Limitations of Surrogate Modeling

Limitations of this type of model include the following: (i) it needs experimental data to train the model; (ii) the descriptors give a reference of relevant parameters to the target property, but they cannot explain the mechanism; (iii) experimental measurements must be performed to validate the predictions; (iv) for new polymers outside of the sublibrary, new descriptors must be generated, which is time consuming due to the need for MD simulations. However, this last limitation is only encountered for the first property that you wish to model, for once the descriptors are generated, they can be used to build predictive models for several properties of the polymer library. The obtained models for water uptake can be improved by increasing the size of training set, by generating more meaningful descriptors, such as 3D descriptors in explicit water, by improving the descriptor selection algorithm, and by identifying other surrogate methods.

## 4. Conclusions

This study describes a new approach to modeling dynamic properties and demonstrates the potential value of this approach. In particular, we developed models for water uptake for a library of polymers using only a small training set and molecular descriptors for all the polymers in the library. We also demonstrate that using a consolidated parameter of water uptake, a dynamic property, gives a more accurate model than using a more conventional approach of all experimental measurement as independent values. By separating time points from one experiment, information about the slope, rate, and progression of the dynamic property is not considered in the model. And, since data points are not independent, accuracy of predictions is compromised.

A surrogate model was built to accurately predict equilibrium water uptake of a polymer sublibrary of 56 L-tyrosine-derived polyarylates using a small training set and only three descriptors selected from a large set of descriptors, calculated from either 2D or 3D structures. Those descriptors included atom counts; 3D information about electron diffraction (3D-MoRSE); and chemical properties of molecular atoms, branching, cyclicity, and conformational changes (GETAWAY). Although these descriptors can be used only for this model in this polymer library, the methodology for selecting descriptors can be applied to any polymer library and/or polymer property.

The model was able to accurately predict low, intermediate, and high levels of water uptake for up to 12 of the 18 polymers. Using this model, predictions were obtained for the rest of the sublibrary. Those predictions can be used primarily as a reference of order of magnitude and ranking of polymers in terms of water uptake.

Finally, having several semiempirical models for different polymer properties such as glass transition temperature, contact angle, fibrinogen adsorption, cell response, water uptake, and degradation for the same polymer library may be used to select a polymer for a specific application. With a known set of design criteria, a group of polymers can be selected from the mentioned models. After this selection, the actual parameters must be measured experimentally, the models must be validated, and the best polymer can be selected to begin the device development process. With this, surrogate modeling of polymer properties may accelerate the discovery and selection of rationally designed materials for a target application.

## Figures and Tables

**Table 1:** Subset of the library of L-tyrosine-derived polyarylates used in this study.

Polymer^a^	*M*_*w*_ (kDa)^b,c^	*T*_*g*_ (°C)^d^	Polymer set for model	Predictions
Poly(DTO sebacate)	123 ± 1	16	●	
Poly(DTB adipate)	111 ± 3	42	●	
Poly(DTO succinate)	84 ± 6	43	●	
Poly(DTE adipate)	126 ± 7	59	●	
Poly(DTE glutarate)	80 ± 1	64	●	
Poly(DTB succinate)	145 ± 11	67	●	
Poly(HTH sebacate)	64 ± 5	23	●	
Poly(HTH adipate)	87 ± 2	40	●	
Poly(DTM sebacate)	126 ± 4	45	●	
Poly(DTiP adipate)	144 ± 2	55	●	
Poly(DTM adipate)	99 ± 3	67	●	
Poly(HTE succinate)	★	78	●	
Poly(DTO adipate)	132 ± 2	26	●	
Poly(DTsB^*∗*^R(+)methyladipate^*∗*^)	79 ± 3	45	●	
Poly(DTsB^*∗*^ R(+) glutarate)	86 ± 3	46	●	
Poly(DTM R(+) methyladipate^*∗*^)	68 ± 1	53	●	
Poly(DTBn adipate)	69 ± 8	61	●	
Poly(HTE adipate)	37 ± 4	61	●	

Poly(DTO suberate)		21		●
Poly(DTH suberate)		24		●
Poly(HTH suberate)		27		●
Poly(DTO glutarate)		32		●
Poly(DTiB sebacate)		33		●
Poly(DTH R(+) methyladipate^*∗*^)		33		●
Poly(DTH L(−) methyladipate^*∗*^)		33		●
Poly(DTH adipate)		34		●
Poly(DTB R(+) methyladipate^*∗*^)		35		●
Poly(DTB L(−) methyladipate^*∗*^)		35		●
Poly(DTB suberate)		37		●
Poly(DTO diglycolate)		40		●
Poly(DTBn sebacate)		42		●
Poly(DTH glutarate)		43		●
Poly(DTH diglycolate)		45		●
Poly(DTsB^*∗*^ L(−) methyladipate^*∗*^)		45		●
Poly(DTsB^*∗*^ L(−) glutarate)		46		●
Poly(DTsB^*∗*^ R(+) suberate)		46		●
Poly(DTsB^*∗*^ L(−) suberate)		46		●
Poly(DTsB^*∗*^ R(+) adipate)		50		●
Poly(DTsB^*∗*^ L(−) adipate)		50		●
Poly(DTB glutarate)		50		●
Poly(DTH succinate)		53		●
Poly(DTM L(−) methyladipate^*∗*^)		53		●
Poly(HTE suberate)		54		●
Poly(DTiP R(+) methyladipate^*∗*^)		54		●
Poly(DTiP L(−) methyladipate^*∗*^)		54		●
Poly(DTM suberate)		55		●
Poly(DTBn R(+) methyladipate^*∗*^)		55		●
Poly(DTBn L(−) methyladipate^*∗*^)		55		●
Poly(DTiB adipate)		56		●
Poly(DTE R(+) methyladipate^*∗*^)		63		●
Poly(DTE L(−) methyladipate^*∗*^)		63		●
Poly(HTE R(+) methyladipate^*∗*^)		63		●
Poly(HTE L(−) methyladipate^*∗*^)		63		●
Poly(DTB diglycolate)		64		●
Poly(DTiB succinate)		75		●

^a^The “*∗*” symbol indicates the presence of more than one chiral center in the polymer repeat unit.

^b^Molecular weight (*M*_*w*_) was measured by THF-GPC (mean value of three different films ± standard deviation (SD)).

^c^The “★” symbol indicates the polymers that did not dissolve in THF and, thus, *M*_*w*_ could not be measured, and degradation could not be measured.

^d^Glass transition temperature (*T*_*g*_) was measured by DSC for the dry polymer before pressing.

**Table 2:** Equilibrium water uptake for 18 polymers of the L-tyrosine-derived polyarylate library.

Polymer^a^	Equilibrium water uptake (%)
Poly(DTB adipate)	18.2 ± 1.2
Poly(DTB succinate)	4.0 ± 0.3
Poly(DTBn adipate)	32.2 ± 7.2
Poly(DTE adipate)	36.2 ± 3.2
Poly(DTE glutarate)	29.6 ± 3.4
Poly(DTiP adipate)	27.6 ± 1.0
Poly(DTM adipate)	14.5 ± 3.5
Poly(DTM sebacate)	12.3 ± 2.7
Poly(DTO adipate)	6.1 ± 0.3
Poly(DTO sebacate)	2.7 ± 0.4
Poly(DTO succinate)	3.5 ± 0.6
Poly(HTE adipate)	7.8 ± 1.1
Poly(HTE succinate)	43.1 ± 10.6
Poly(HTH adipate)	18.0 ± 2.1
Poly(HTH sebacate)	2.3 ± 0.4
Poly(DTM R(+) methyladipate)	90.1 ± 8.8
Poly(DTsB R(+) glutarate)	97.4 ± 4.1
Poly(DTsB R(+) methyladipate)	136.5 ± 10.0

^a^Polymers are ordered by name used in the descriptor set.

**Table 3:** Best descriptors and their variability within the training set and within the complete set of 56 polymers.

Model	Descriptor	SD for polymers of the model	SD for the complete library
All data points	Hydrophilic factor	0.246	0.212
	SMR_VSA6	0.291	0.242
	GGI3	0.227	0.264
	MATS3m	0.256	0.273
	C-003	0.394	0.478
	G2m vacuum	0.231	0.255

WU_eq_	nCt	0.287	0.316
	Mor25m water	0.212	0.238
	R8p+ vacuum	0.243	0.242

**Table 4:** Summary of models for water uptake.

Model	*n* training set	Number of descriptors	*R* ^2^ training	Within experimental variability (training)	*R* ^2^ cross-validation (10%)	Within experimental variability (test)
All data points	189	6+ time	0.92	30/189 (16%)	0.83	3/18 (17%)
WU_eq_	18	3	0.97	12/18 (67%)	0.78	9/18 (50%)

## References

[1] Kohn J., Welsh W. J., Knight D. (2007). A new approach to the rationale discovery of polymeric biomaterials. *Biomaterials*.

[2] Lantry J. M., Roberts C. S., Giannoudis P. V. (2008). Operative treatment of scapular fractures: a systematic review. *Injury*.

[3] Griffith L. G. (2000). Polymeric biomaterials. *Acta Biomaterialia*.

[4] Angelova N., Hunkeler D. (1999). Rationalizing the design of polymeric biomaterials. *Trends in Biotechnology*.

[5] Shoichet M. S. (2010). Polymer scaffolds for biomaterials applications. *Macromolecules*.

[6] Kranz H., Ubrich N., Maincent P., Bodmeier R. (2000). Physicomechanical properties of biodegradable poly(D,L-lactide) and poly(D,L-lactide-co-glycolide) films in the dry and wet states. *Journal of Pharmaceutical Sciences*.

[7] Hopkins C., McHugh P. E., O'Dowd N. P., Rochev Y., McGarry J. P. (2013). A combined computational and experimental methodology to determine the adhesion properties of stent polymer coatings. *Computational Materials Science*.

[8] Ahlneck C., Zografi G. (1990). The molecular basis of moisture effects on the physical and chemical stability of drugs in the solid state. *International Journal of Pharmaceutics*.

[9] Caccavo D., Cascone S., Lamberti G., Barba A. A. (2015). Modeling the drug release from hydrogel-based matrices. *Molecular Pharmaceutics*.

[10] Tanaka M., Hayashi T., Morita S. (2013). The roles of water molecules at the biointerface of medical polymers. *Polymer Journal*.

[11] Valenzuela L. M., Michniak B., Kohn J. (2011). Variability of water uptake studies of biomedical polymers. *Journal of Applied Polymer Science*.

[12] Kholodovych V., Gubskaya A. V., Bohrer M. (2008). Prediction of biological response for large combinatorial libraries of biodegradable polymers: polymethacrylates as a test case. *Polymer*.

[13] Webster D. C., Meier M. A. R. (2010). Polymer libraries: preparation and applications. *Polymer Libraries*.

[14] Costache A. D., Ghosh J., Knight D. D., Kohn J. (2010). Computational methods for the development of polymeric biomaterials. *Advanced Engineering Materials*.

[15] Gubskaya A. V., Matta C. F. (2010). Quantum-chemical descriptors in QSAR/QSPR modeling: achievements, perspectives and trends. *Quantum Biochem*.

[16] Le T., Epa V. C., Burden F. R., Winkler D. A. (2012). Quantitative structure-property relationship modeling of diverse materials properties. *Chemical Reviews*.

[17] Serna F., García F., De La Peña J. L., García J. M. (2008). Aromatic polyisophthalamides with mononitro, dinitro and trinitroiminobenzoyl pendant groups. *High Performance Polymers*.

[18] Crank J. (1975). *The Mathematics of Diffusion*.

[19] Roy S., Xu W. X., Park S. J., Liechti K. M. (1999). Anomalous moisture diffusion in viscoelastic polymers: modeling and testing. *Journal of Applied Mechanics*.

[20] Loh W. K., Crocombe A. D., Wahab M. M. A., Ashcroft I. A. (2005). Modelling anomalous moisture uptake, swelling and thermal characteristics of a rubber toughened epoxy adhesive. *International Journal of Adhesion and Adhesives*.

[21] Weibull W. (1951). A statistical distribution of wide applicability. *Journal of Applied Mechanics*.

[22] Popineau S., Rondeau-Mouro C., Sulpice-Gaillet C., Shanahan M. E. R. (2005). Free/bound water absorption in an epoxy adhesive. *Polymer*.

[23] Joannès S., Mazé L., Bunsell A. R. (2014). A simple method for modeling the concentration-dependent water sorption in reinforced polymeric materials. *Composites Part B: Engineering*.

[24] Noorjahan A., Choi P. (2015). Effect of free volume redistribution on the diffusivity of water and benzene in poly(vinyl alcohol). *Chemical Engineering Science*.

[25] Tamai Y., Tanaka H., Nakanishi K. (1996). Molecular dynamics study of polymer-water interaction in hydrogels. 2. Hydrogen-bond dynamics. *Macromolecules*.

[26] Gubskaya A. V., Kusalik P. G. (2004). Molecular dynamics simulation study of ethylene glycol, ethylenediamine, and 2-aminoethanol. 1. The local Structure in pure liquids. *Journal of Physical Chemistry A*.

[27] Behler J., Price D. W., Drew M. G. B. (2001). Water structuring properties of carbohydrates, molecular dynamics studies on 1,5-anhydro-D-fructose. *Physical Chemistry Chemical Physics*.

[28] Canales M., Aradilla D., Alemán C. (2011). Water absorption in polyaniline emeraldine base. *Journal of Polymer Science Part B: Polymer Physics*.

[29] Xiang T.-X., Anderson B. D. (2005). Distribution and effect of water content on molecular mobility in poly(vinylpyrrolidone) glasses: a molecular dynamics simulation. *Pharmaceutical Research*.

[30] Xiang T. X., Anderson B. D. (2014). Water uptake, distribution, and mobility in amorphous poly(d,l-lactide) by molecular dynamics simulation. *Journal of Pharmaceutical Sciences*.

[31] Kholodovych V., Smith J. R., Knight D., Abramson S., Kohn J., Welsh W. J. (2004). Accurate predictions of cellular response using QSPR: a feasibility test of rational design of polymeric biomaterials. *Polymer*.

[32] Smith J. R., Kholodovych V., Knight D., Welsh W. J., Kohn J. (2005). QSAR models for the analysis of bioresponse data from combinatorial libraries of biomaterials. *QSAR & Combinatorial Science*.

[33] Smith J. R., Kholodovych V., Knight D., Kohn J., Welsh W. J. (2005). Predicting fibrinogen adsorption to polymeric surfaces in silico: a combined method approach. *Polymer*.

[34] Smith J. R., Seyda A., Weber N., Knight D., Abramson S., Kohn J. (2004). Integration of combinatorial synthesis, rapid screening, and computational modeling in biomaterials development. *Macromolecular Rapid Communications*.

[35] Smith J. R., Knight D., Kohn J. (2004). Using surrogate modeling in the prediction of fibrinogen adsorption onto polymer surfaces. *Journal of Chemical Information and Computer Sciences*.

[36] Gubskaya A. V., Kholodovych V., Knight D., Kohn J., Welsh W. J. (2007). Prediction of fibrinogen adsorption for biodegradable polymers: integration of molecular dynamics and surrogate modeling. *Polymer*.

[37] Ghosh J., Lewitus D. Y., Chandra P. (2011). Computational modeling of in vitro biological responses on polymethacrylate surfaces. *Polymer*.

[38] Cranford S. W., de Boer J., van Blitterswijk C., Buehler M. J. (2013). Materiomics: an -omics approach to biomaterials research. *Advanced Materials*.

[39] Le T. C., Conn C. E., Burden F. R., Winkler D. A. (2013). Computational modeling and prediction of the complex time-dependent phase behavior of lyotropic liquid crystals under in meso crystallization conditions. *Crystal Growth and Design*.

[40] Gubskaya A. V., Khan I. J., Valenzuela L. M., Lisnyak Y. V., Kohn J. (2013). Investigating the release of a hydrophobic peptide from matrices of biodegradable polymers: an integrated method approach. *Polymer*.

[41] Brocchini S., James K., Tangpasuthadol V., Kohn J. (1997). A combinatorial approach for polymer design. *Journal of the American Chemical Society*.

[42] Hooper K. A., Macon N. D., Kohn J. (1998). Comparative histological evaluation of new tyrosine-derived polymers and poly (L-lactic acid) as a function of polymer degradation. *Journal of Biomedical Materials Research*.

[43] Bloom H. L., Constantin L., Dan D. (2011). Implantation success and infection in cardiovascular implantable electronic device procedures utilizing an antibacterial envelope. *Pacing and Clinical Electrophysiology*.

[44] Fiordeliso J., Bron S., Kohn J. (1994). Design, synthesis, and preliminary characterization of tyrosine-containing polyarylates: new biomaterials for medical applications. *Journal of Biomaterials Science*.

[45] Valenzuela L. M., Zhang G., Flach C. R. (2012). Multiscale analysis of water uptake and erosion in biodegradable polyarylates. *Polymer Degradation and Stability*.

[46] Witten I. H., Frank E. (2000). *Data Mining: Practical Machine Learning Tools and Techniques with JAVA Implementations*.

[47] Chemical Computing Group (2003). *MOE (The Molecular Operating Environment)*.

[48] Todeschini R., Consonni V., Mauri A., Pavan M. (2003). *Dragon Web Version, 3.0*.

[49] Schrödinger (2005). *Schrödinger Release 2005: MacroModel, V. 8.5*.

[50] Still W. C., Tempczyk A., Hawley R. C., Hendrickson T. (1990). Semianalytical treatment of solvation for molecular mechanics and dynamics. *Journal of the American Chemical Society*.

[51] Jorgensen W. L., Maxwell D. S., Tirado-Rives J. (1996). Development and testing of the OPLS all-atom force field on conformational energetics and properties of organic liquids. *Journal of the American Chemical Society*.

[52] Toropova A. P., Toropov A. A., Kudyshkin V. O., Leszczynska D., Leszczynski J. (2014). Optimal descriptors as a tool to predict the thermal decomposition of polymers. *Journal of Mathematical Chemistry*.

[53] Duchowicz P. R., Fioressi S. E., Bacelo D. E., Saavedra L. M., Toropova A. P., Toropov A. A. (2015). QSPR studies on refractive indices of structurally heterogeneous polymers. *Chemometrics and Intelligent Laboratory Systems*.

[54] Hall M. A. (1999). *Correlation-Based Feature Selection for Machine Learning*.

[55] Quinlan J. R. (1993). *Programs for Machine Learning*.

[56] Todeschini R., Bettiol C., Giurin G., Gramatica P., Miana P., Argese E. (1996). Modeling and prediction by using WHIM descriptors in QSAR studies: Submitochondrial particles (SMP) as toxicity biosensors of chlorophenols. *Chemosphere*.

[57] Afantitis A., Melagraki G., Makridima K., Alexandridis A., Sarimveis H., Iglessi-Markopoulou O. (2005). Prediction of high weight polymers glass transition temperature using RBF neural networks. *Journal of Molecular Structure*.

[58] Seyhan A. T., Tayfur G., Karakurt M., Tanoglu M. (2005). Artificial neural network (ANN) prediction of compressive strength of VARTM processed polymer composites. *Computational Materials Science*.

[59] Gao J. W., Wang X. Y., Li X. B., Yu X., Wang H. (2006). Prediction of polyamide properties using quantum-chemical methods and BP artificial neural networks. *Journal of Molecular Modeling*.

[60] Liu W. Q., Yi P. G., Tang Z. L. (2006). QSPR models for various properties of polymethacrylates based on quantum chemical descriptors. *QSAR & Combinatorial Science*.

[61] Gharagheizi F. (2007). QSPR analysis for intrinsic viscosity of polymer solutions by means of GA-MLR and RBFNN. *Computational Materials Science*.

[62] Xu J., Liang H., Chen B., Xu W., Shen X., Liu H. (2008). Linear and nonlinear QSPR models to predict refractive indices of polymers from cyclic dimer structures. *Chemometrics and Intelligent Laboratory Systems*.

[63] Todeschini R., Consonni V. (2000). *Handbook of Molecular Descriptors*.

[64] Wildman S. A., Crippen G. M. (1999). Prediction of physicochemical parameters by atomic contributions. *Journal of Chemical Information and Computer Sciences*.

[65] Xia J.-F., Han K., Huang D.-S. (2010). Sequence-based prediction of protein-protein interactions by means of rotation forest and autocorrelation descriptor. *Protein and Peptide Letters*.

[66] Todeschini R., Gramatica P. (1998). New 3D molecular descriptors: the WHIM theory and QSAR applications. *Perspectives in Drug Discovery and Design*.

[67] Consonni V., Todeschini R., Pavan M. (2002). Structure/response correlations and similarity/diversity analysis by GETAWAY descriptors. 1. Theory of the novel 3D molecular descriptors. *Journal of Chemical Information and Computer Sciences*.

[68] Gasteiger J., Schuur J., Selzer P., Steinhauer L., Steinhauer V. (1997). Finding the 3D structure of a molecule in its IR spectrum. *Fresenius' Journal of Analytical Chemistry*.

[69] Liu W. (2010). Prediction of glass transition temperatures of aromatic heterocyclic polyimides using an ANN model. *Polymer Engineering and Science*.

[70] Mattioni B. E., Jurs P. C. (2002). Prediction of glass transition temperatures from monomer and repeat unit structure using computational neural networks. *Journal of Chemical Information and Computer Sciences*.

[71] Jelcic Z. (2004). Solvent molecular descriptors on poly(d, l-lactide-co-glycolide) particle size in emulsification—diffusion process. *Colloids and Surfaces A: Physicochemical and Engineering Aspects*.

[72] Gubskaya A. V., Bonates T. O., Kholodovych V. (2011). Logical analysis of data in structure-activity investigation of polymeric gene delivery. *Macromolecular Theory and Simulations*.

